# Prevalence of extended-spectrum β-lactamases, AmpC, and carbapenemases in *Proteus mirabilis* clinical isolates

**DOI:** 10.1186/s12866-022-02662-3

**Published:** 2022-10-11

**Authors:** Mona Shaaban, Soha Lotfy Elshaer, Ola A. Abd El-Rahman

**Affiliations:** 1 Department of Microbiology and Immunology, Faculty of Pharmacy, Mansoura University, Mansoura, 35516 Egypt; 2 Department of Microbiology and Immunology, Faculty of Pharmacy (Girls), Al-Azhar University, Cairo, 11651 Egypt

**Keywords:** *Proteus mirabilis*, Extended-spectrum β-lactamase, AmpC β-lactamase, Carbapenemase, ERIC-PCR

## Abstract

**Background:**

*Proteus mirabilis* is an opportunistic pathogen, causing a variety of community-acquired and nosocomial illnesses. It poses a potential threat to patients via the production of β-lactamases, which decrease the efficacy of antimicrobial treatment and impair the management of its pathogenicity. Hence, this study was established to determine the prevalence of extended-spectrum β-lactamases (ESBLs), AmpC, and carbapenemases of *P. mirabilis* isolated from various clinical specimens.

**Results:**

*Proteus mirabilis* was identified in 20.7% (58/280) of specimens. ESBL producers were present at a rate of 51.7% (30/58). All AmpC-positive isolates (*n* = 20) produced ESBLs as well, so 66.7% of ESBL-producing isolates coproduced AmpC enzymes. The modified Hodge test confirmed carbapenemase production in six out of seven imipenem nonsusceptible isolates. Of these, only two (5.7%) isolates were also ESBL-and AmpC-positive. Antibiotic resistance reached the highest level for cotrimoxazole (62.1%, *n* = 36/58 isolates) and the lowest for imipenem (12.1%, *n* = 7/58 isolates). The levels of multidrug-resistant (MDR) was 41.4% among the tested isolates. The *bla*_SHV_ (83.3%), *bla*_AmpC_ (80%), and *bla*_VIM-1_ (50%) were the most detected genes in phenotypically confirmed ESBL-, AmpC-, and carbapenemase-producing isolates, respectively. Besides, more than a half of the tested *P. mirabilis* strains (53%) coproduced ESBLs and AmpC. Moreover, two isolates coproduced ESBLs and AmpC together with carbapenemases. Furthermore, dendrogram analysis showed great genetic divergence based on the 21 different enterobacterial repetitive intergenic consensus (ERIC) patterns (P1–P21) through the 34 β-lactamase producers. ERIC analysis distinguished clonal similarities between isolates 21 and 22 in P2 and 9 and 10 in P4, which were isolated from the same clinical source and possessed similar patterns of β-lactamase-encoding genes.

**Conclusion:**

Hence, there is an urgent need to monitor hospitalized patients and improve healthcare in order to reduce the incidence of infection and outbreaks of infection with antibiotic-resistant *Proteus*.

**Supplementary Information:**

The online version contains supplementary material available at 10.1186/s12866-022-02662-3.

## Background

*Proteus mirabilis* is an enterobacterial species that naturally colonizes the gastrointestinal lumen and is present in many environmental features, such as water, soil, and feces-contaminated material [1, 2]. It is an opportunistic pathogen responsible for serious infections of the human urinary tract, respiratory tract, wounds, otitis media, and blood. Flagella mediate its swarming mobility on the surface of solid media, so it can easily reach the kidney and invade the urinary tract. Hence, *P. mirabilis* is one of the most common causative agents of urinary tract infections (UTIs), particularly in catheterized patients [3]. Biofilm structures of *P. mirabilis* play a critical role in protecting it against antibiotics as well as host defense mechanisms and cause challenges in treatment as a result of conferring a multidrug-resistant (MDR) or extensively drug-resistant (XDR) status [4].

β-lactam resistance, mediated by the synthesis of β-lactamases, is being more and more frequently reported among *P. mirabilis*. Extended-spectrum β-lactamases (ESBLs), AmpC, and carbapenemases are the most common β-lactamase enzymes [5]. Infections caused by ESBL-producing isolates are a serious international problem, causing significant increases in morbidity and mortality among hospitalized patients. In addition, ESBL producers exhibit coresistance with other different antibiotic classes such as quinolones, aminoglycosides, and sulfa drugs [6]. Formerly, *bla*_SHV_ and *bla*_TEM_ were the predominant ESBL genotypes found in *Enterobacteriaceae* isolates, but recently *bla*_CTX-M_ (especially *bla*_CTX-M-15_) has become the most common genotype [7].

AmpC, another variant of β-lactamases, is unlike ESBLs not affected by either cephamycins or β-lactamase inhibitors [8]. The AmpC enzyme may mask the effect of ESBLs and their recognition, so it is very complicated to treat their coexistence in the same isolate. Thus, AmpC-producing isolates act as a silent reservoir for ESBLs [9]. Carbapenems are the remaining treatment option against serious ESBL- and AmpC-related infections [10]. Unfortunately, treatment failure is observed due to the rapid propagation of carbapenem-resistant isolates [11]. Changes in the porin channel in addition to coexpression of AmpC, ESBL, or carbapenemase enzymes are the most common mechanisms of carbapenem resistance in *Enterobacteriaceae* [12–14]. The extensive resistance of Gram-negative bacteria is associated with the transfer of resistance genes via transferable genetic elements such as plasmids, which can readily pass through mutant clones and spread rapidly between countries. Most of this spread is therefore undetected as the normal human flora acquires those resistance genes and becomes a silent source of endogenous infections [15]. In view of the increasing prevalence of *Proteus* resistance to various antimicrobials, especially β-lactam antibiotics, the objective of this study is to detect mechanisms of resistance to β-lactams (i.e., ESBLs, AmpC, and carbapenemases) among *P. mirabilis* isolates collected from healthcare facilities using phenotypic and molecular testing, to support the potential therapeutic options for treating these complicated clinical infections. Then, we determined the genetic diversity of different β-lactamase-producing *P. mirabilis* isolates using enterobacterial repetitive intergenic consensus-polymerase chain reaction (ERIC-PCR).

## Methods

### Bacterial isolates and growth media

A total of 280 nonduplicate Gram-negative isolates were purified from different clinical samples such as urine, wounds, blood, sputum, and cerebrospinal fluid (CSF) from Aug to Dec 2021. The samples were collected from patients ≥ 18 years. The clinical specimens were collected from Al-Qasr Al*-*Aini University Hospitals with approval from the ethics committee of the Faculty of Pharmacy, Al-Azhar University (Protocol code 306 at 23/8/2021). *Proteus mirabilis* was provisionally identified based on characteristic growth on blood agar, non-lactose-fermenting colonies on MacConkey’s agar media (Oxoid, UK), and various biochemical reactions [16–18], and were confirmed using the automated Vitek 2 system (bioMérieux, Inc., Hazelwood, MO, USA). The purified isolates were preserved at − 80 °C in glycerol (25% v/v).

### Antimicrobial susceptibility test

A routine antimicrobial susceptibility test was performed by the Kirby–Bauer disk diffusion method against all *P. mirabilis* isolates on Mueller Hinton agar (MHA; Oxoid, UK) and the results were interpreted in accordance with the Clinical and Laboratory Standards Institute [19] criteria. The antibiotics (Oxoid, UK) used were piperacillin (PRL, 100 μg), amoxicillin/clavulanic acid (AMC, 20/10 μg), aztreonam (ATM, 30 μg), imipenem (IPM, 10 μg), cefoxitin (FOX, 30 μg), ceftazidime (CAZ, 30 μg), cefotaxime (CTX, 30 μg), ciprofloxacin (CIP, 5 μg), cotrimoxazole (TS, 25 μg), gentamicin (GM, 10 μg), and amikacin (AK, 30 μg). Resistance to three or more classes of antimicrobial agents was defined as MDR [20].

The multiple antibiotic resistance index (MARI) was calculated by dividing the sum of antibiotics against which the bacterial species displayed resistance by the total number of antibiotics to which the isolates were subjected [21].

### Phenotypic detection of β-lactamases

#### Detection of extended-spectrum β-lactamases (ESBLs)

A double-disk synergy test (DDST) was performed to examine the release of ESBL enzymes. The surface of MHA plates was dried and streaked with overnight cultures of the tested isolates diluted to 0.5 MacFarland. Ceftazidime (30 μg) and cefotaxime (30 μg) disks were applied on the agar surface 15 mm away from the centered amoxicillin–clavulanic acid (20/10 μg) disk and the plates were incubated at 37 °C for 24 h. Positive production of ESBL enzymes was detected by clear enhancement in the inhibitory zones around any of the expanded-spectrum cephalosporin disks toward amoxicillin–clavulanic acid, and denoted as “champagne-cork” or “keyhole” [22]. In a DDST with AmpC-positive isolates, cloxacillin (200 µg/ml) was added to the sterilized melted agar medium, at 45 °C, to act as an AmpC-type β-lactamase inhibitor [23].

#### Screening for AmpC β-lactamase-producing isolates by inhibitor-based test

Cefoxitin-cloxacillin DDST (CC-DDS) was conducted based on the inhibitory effect of cloxacillin on AmpC enzyme production. A disk of cefoxitin (30 μg) only and another supplemented with 20 mg of cloxacillin were placed on MHA plate inoculated with a bacterial suspension of 0.5 McFarland standards and incubated overnight at 37 °C. Any increase in the size of the inhibitory zone by ≥ 4 mm for cefoxitin/cloxacillin compared with that for the unsupplemented cefoxitin disk was considered to indicate AmpC production [24].

#### Detection of carbapenemase enzyme

A modified Hodge test (MHT) was performed to confirm the release of carbapenemases from *P. mirabilis* isolates in accordance with CLSI guidelines [25]. *Escherichia coli* ATCC 25922 was cultured overnight in peptone water to 0.5 McFarland opacity standards and swabbed onto MHA plate. A meropenem disk (10 µg) was placed at the center of the plate and the test isolates were streaked as a thin straight line from the edge of the disk to the plate edge. The plates were incubated in an inverted position at 37 °C overnight. The presence of a distorted inhibitory zone (clover-leaf shape) of *E. coli* ATCC 25922 growth toward the meropenem disk was considered to indicate a positive test result [26].

### Genetic detection of β-lactamases

Phenotypically confirmed ESBL-, AmpC, and carbapenemase-positive *Proteus* isolates were subjected to PCR using specific primers for ESBL genes (*bla*_TEM,_*bla*_SHV_, *bla*_CTX-2_, *bla*_CTX-M_), AmpC-encoding genes (*bla*_AmpC,_*bla*_ACT_, *bla*_ACC_, *bla*_FOX_), and carbapenemase genes (*bla*_KPC_, *bla*_IMP_, *bla*_NDM_, *bla*_VIM-1_, *bla*_VIM-2_, and *bla*_OXA_) (Supplementary Table 1). The amplification cycles included initial denaturation at 94 °C for 3 min, followed by 35 cycles of denaturation at 94 °C for 30 s, annealing according to the temperature specified in Supplementary Table 1 for 30 s, and extension at 72 °C for 60 s; the reaction was then ended by a final extension step at 72 °C for 10 min. A negative control (molecular grade RNase free water) was included in all PCR assays.

### Molecular typing of *P. mirabilis* isolates by enterobacterial repetitive intergenic consensus-PCR (ERIC-PCR)

*P. mirabilis* isolates harboring one or more β-lactamase-encoding genes as determined by PCR were fingerprinted by ERIC-PCR using the following primer pair: ERIC 1 (5′-ATGTAAGCTCCTGGGGATTCAC-3′) and ERIC 2 (5′-AAGTAAGTGACTGGGGTGAGCG-3′). The DNA amplification process was performed in a volume of 50 μL, including 2 U Go Taq DNA polymerase (Fermatas), 2.5 mM MgCl_2_ (Invitrogen), 0.25 mM each deoxynucleotide triphosphate, 100 ng of DNA template, and 0.5 μM primers, while the remaining volume was filled up with PCR-grade water. The PCR program used was as follows: denaturation at 95 °C for 5 min, followed by 35 cycles of denaturation at 94 °C for 30 s, annealing at 42 °C for 40 s, and extension at 72 °C for 5 min, and then a single final stage at 72 °C for 10 min [27].

### Dendrogram and phylogenetic relationships

The banding pattern of PCR product was analyzed on a 1.5% agarose gel along with a negative control (without DNA template). The dendrogram was created by the unweighted pair group method with arithmetic mean (UPGMA) using the DendroUPGMA server (http://genomes.urv.cat/UPGMA) [28]. It was constructed using the Dice coefficient with a 1% tolerance limit and 1% optimization. Cluster relatedness of collected isolates with ≥ 70% similarity was considered to indicate an identical pattern type [29].

### Statistical analysis

Comparison of proportions and graphic were performed with GraphPad Prism software (version 5.01) and Microsoft Excel (Microsoft Cooperation, 2010). Fisher’s exact and chi-square tests were used to determine the difference in resistance between β-lactamase positive and β-lactamase negative isolates. Moreover, the correlation between β-lactamase encoding genes was carried out using R statistical platform (https:// www.r- project. org) in R-studio version 1.4.1106, using the Spearman’s rank correlation and strength of the association was expressed as Spearman’s correlation coefficient (*rs*) between − 1 and + 1. For all statistical analyses, *P values* < 0.05 were considered to indicate a statistically significant difference.

## Results

### Identification of clinical isolates

Out of 280 clinical Gram-negative isolates processed, 58 *P. mirabilis* isolates were identified. The majority of the isolates were obtained from urine (69%, 40/58) followed by wounds (13.8%, 8/58), blood (6.9%, 4/58), sputum (6.9%, 4/58), and CSF (3.4%, 2/58).

### Antimicrobial resistance pattern

The resistance pattern of the tested 4 antimicrobial categories including 11 antimicrobial agents against all *P. mirabilis* was clarified in Table 1. The resistance to cotrimoxazole, gentamicin, cefoxitin and ceftazidime was highly prevalent (60.3%, 37.9%, 36.2% and 32.8%, respectively). Whereas the resistance rate to piperacillin, amoxicillin-clavulanic acid, cefotaxime, ciprofloxacin and amikacin were lower than 30%. The highest sensitivity was observed for imipenem and aztreonam as 87.9% and 84.5%, respectively.

**Table 1:** Antibiogram results of all *Proteus mirabilis* isolates

Antibiotics	Total (*n* = 58 isolates)					
	**(R) No**	**(R) %**	**(I) No**	**(I) %**	**(S) No**	**(S) %**
**Piperacillin**	13	22.4	5	8.6	40	69.0
**Amoxicillin/clavulanic acid**	13	22.4	13	22.4	32	55.2
**Aztreonam**	8	13.8	1	1.7	49	84.5
**Imipenam**	1	1.7	6	10.3	51	87.9
**Cefoxitin**	21	36.2	7	12.1	30	51.7
**Cefotaxime**	17	29.3	7	12.1	34	58.6
**Ceftazidime**	19	32.8	5	8.6	34	58.6
**Ciprofloxacin**	15	25.9	6	10.3	37	63.8
**Cotrimoxazole**	35	60.3	1	1.7	22	37.9
**Gentamicin**	22	37.9	1	1.7	35	60.3
**Amikacin**	12	20.7	10	17.2	36	62.1

*R* Resistant, *I* Intermediate, *S* Sensitive, *N* Number of isolates

A total of 17 isolates were considered as MDR, from which 4 (23.5%) and 13 (76.5%) isolates belonged to 4 and 3 classes of antibiotics, respectively (Table 2). Among MDR isolates, 52.9% were isolated from urinary tract, 23.5% were from blood and 11.8% were from each wound and sputum. The MDR isolates showed 12 antibiotic resistance patterns to 3–9 antibiotics and including 8 unique patterns. The MARI ranged from 0.27 to 0.82 amongst all MDR *Proteus* isolates that are presented in Table 2.

**Table 2:** Different antibiotic resistance phenotypes and indices in 17 MDR *P. mirabilis* isolates

Antibiotic profile	No. of AB groups	No of phenotypes (%)	No. of AB showing resistance	MARI
PRL/AMC/ATM/FOX/CTX/CAZ/CIP/TS/AK	4	1 (5.9%)	9	0.82
PRL/AMC/ATM/CTX/CAZ/CIP/TS/GM/AK	4	2 (11.8%)	9	0.82
PRL/AMC/FOX/CTX/CAZ/CIP/TS/GM/AK	4	1 (5.9%)	9	0.82
PRL/AMC/FOX/CIP/TS/GM	4	1 (5.9%)	6	0.55
PRL/FOX/CTX/CAZ/CIP/TS/GM/AK	4	1 (5.9%)	8	0.73
PRL/CTX/CAZ/CIP/TS/GM/AK	4	2 (11.8%)	7	0.64
PRL/CTX/CAZ/CIP/GM/AK	4	1 (5.9%)	6	0.55
AMC/FOX/TS/AK	4	1 (5.9%)	4	0.36
ATM/FOX/CTX/CAZ/CIP/TS/GM/AK	4	3 (17.6%)	8	0.73
AMC/FOX/CTX/TS/GM	3	1 (5.9%)	5	0.45
AMC/TS/GM	3	2 (11.8%)	3	0.27
PRL/ TS/GM	3	1 (5.9%)	3	0.27

*PRL* Piperacillin (100 μg), *AMC* Amoxicillin/clavulanic acid (30 μg), *ATM* Aztreonam (30 μg), *IPM* Imipenam (10 μg), *FOX* Cefoxitin (30 μg), *CTX* Cefotaxime, *CAZ* Ceftazidime (30 μg), *CIP* Ciprofloxacin (5 μg), *TS* Cotrimoxazole (25 μg), *GM* Gentamicin (10 μg) and *AK* Amikacin (30 μg), *AB* Antibiotic, *MARI* Multiple antibiotic resistance index

### Phenotypic detection of ESBLs, AmpC, and carbapenemases

**Fig. 1:**
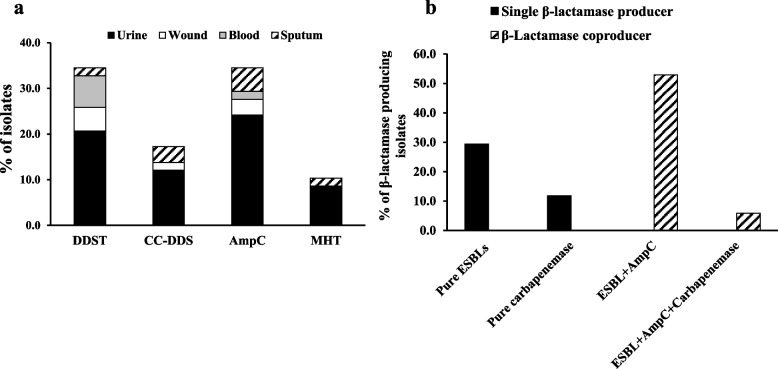
The percent of *P. mirabilis* isolates producing β-lactamase enzymes (**a**): phenotypic detection of β-lactamases enzymes among isolates from different clinical sources (**b**): the prevalence rate of various β-lactamases enzymes among β-lactamases producing isolates. DDST: double disc synergism test, CC-DDS: cefoxitin-cloxacillin double disk synergy test and MHT: Modified Hodge Test

Among the 58 isolates included in this study, 34 (58.6%) were β-lactamase producers. Twenty isolates (34.5%) were ESBL producers, as determined by DDST test (Fig. 1a, and Supplementary Figure S1). Twenty isolates (34.5%) were positive for AmpC β-lactamase, showing an increase in the inhibitory zone on the cefoxitin/cloxacillin disk by ≥ 4 mm relative to the inhibitory zone on the disk with cefoxitin alone (Fig. 1a, and Supplementary Figure S2).

Among AmpC producing isolates, ten isolates (17.2%) ((urine (*n* = 7, 1.1%), wound (*n* = 1, 1.7%) and sputum (*n* = 2, 3.4%)), showed positivity for ESBLs. Hence, the total number of ESBL producers was 30 isolates (51.7%) (Fig. 1b, and Supplementary Figure S3).

Seven isolates (4, 5, 8, 15, 16, 18, and 49) were not susceptible to imipenem, so were considered as putative carbapenemase producers and thus were subjected to confirmatory MHT. MHT showed that 10.3% (6/ 58 isolates) revealed clover leaf-like growth around the meropenem disk and were assigned as positive carbapenemase-producing isolates (Fig. 1a, Supplementary Figure S4). The majority of β-lactamase producers were isolated from urine (23/34 isolates, 67.6%), while *P. mirabilis* isolated from CSF was negative for all detected β-lactamase enzymes.

Among 34 β-lactamase producers, 14 isolates produced only single type of β-lactamases; among those 10 isolates (29.4%) were pure ESBL producers and the remaining 11.8% of isolates were pure carbapenemase producers. On the other hand, 20 isolates produced more than one type of β-lactamases as most of them (18 isolates, 52.9%) coproduced ESBLs and AmpC, whereas only two isolates (5.9%) coproduced ESBLs and AmpC together with carbapenemases (Fig. 1b**)**.

### Antimicrobial susceptibility profile and β-lactamases production

**Fig. 2:**
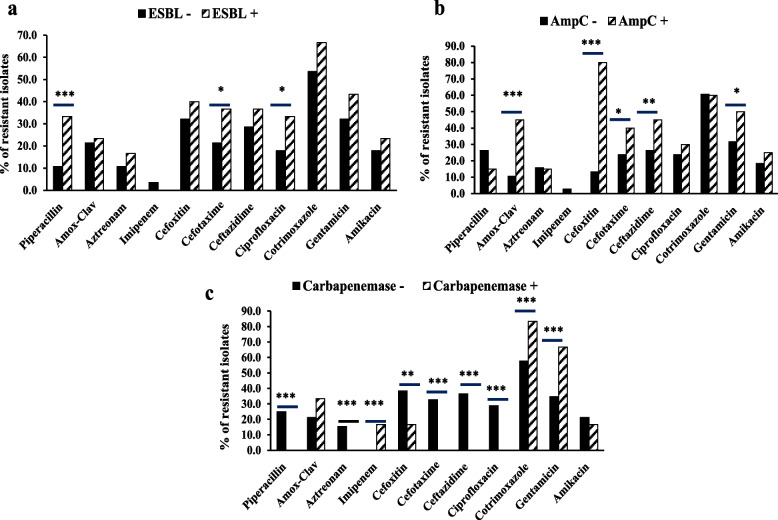
Resistance percentages among β-lacatmase negative (solid columns) and β-lacatmase positive (dashed columns) of *P. mirabilis* to 11 antibiotics for (**a**): ESBL, (**b**): AmpC and (**c**): carbapenemase enzymes. (* significant: *p* < 0.05, ** significant: *p* < 0.01 and ** *significant: *p* < 0.001). Statistical analysis was performed by Fisher Exact method

The relation of antibiotic resistance pattern between β-lactamase-producing positive and negative *P. mirabilis* isolates was clarified in Fig. 2. All ESBL producing isolates had high rates of resistance toward cotrimoxazole, while being susceptible to imipenem. Significantly, the rates of resistance to piperacillin (33.3% vs. 10.7%), cefotaxime (36.7% vs. 21.4%) and ciprofloxacin (33.3% vs. 17.9%) were higher in ESBL + isolates than in ESBL- isolates (Fig. 2a).

Regarding AmpC production (Fig. 2b), the resistance rate in AmpC + was the highest toward cefoxitin and was the lowest toward imipenem than AmpC – isolates. With the exception of piperacillin, aztronam, imipenem, ciprofloxacin and cotrimoxazole, AmpC production had a significant effect on *P. mirabilis* resistance to other antibiotics tested.

Concerning carbapenemases, resistance to cotrimoxazole (83.3%) gentamicin (66.7%) and imipenem (16.7%) was significantly higher in carbapenemase-positive isolates than carbapenemase-negative ones (Fig. 2c)**.**

### Molecular detection of β-lactamases

Thirty isolates positive for ESBLs as determined using the DDST method, with or without cloxacillin, were assessed for genes encoding ESBLs (*bla*_TEM_, *bla*_SHV*,*_* bla*_CTX-2*,*_* bla*_CTX-M_) by PCR. All of those isolates, except isolate (No. 41), contained at least one of these ESBL encoding gene. Among ESBL-encoding genes, the *bla*_SHV_ gene was the most common (*n* = 25, 83.3%), followed by *bla*_CTX-M_ (*n* = 24, 80%) and *bla*_TEM_ (*n* = 22, 73.3%), whereas *bla*_CTX-2_ was the least common (*n* = 4, 13.3%) (Supplementary Table 2).

**Fig. 3:**
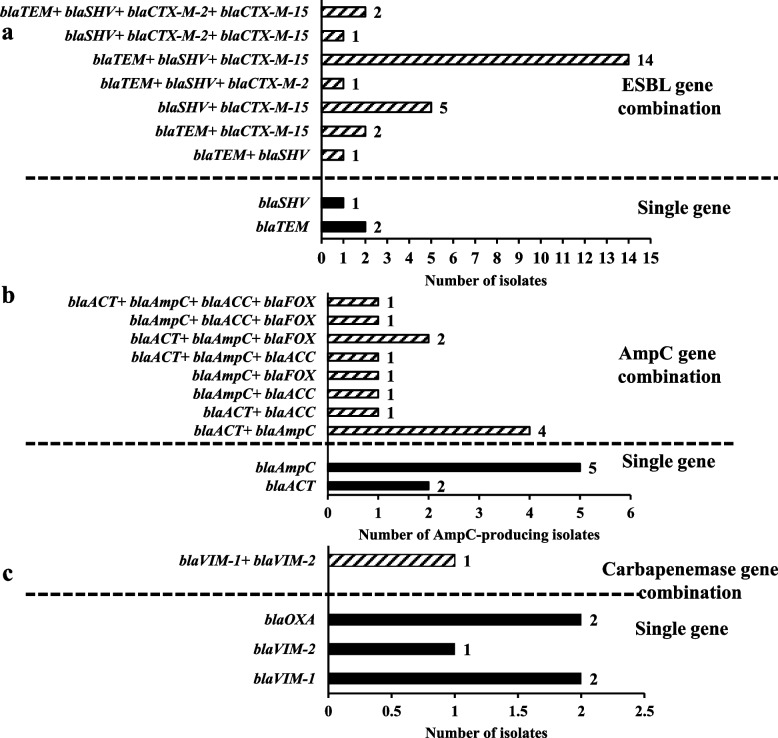
A bar graph illustrating the number of isolates-producing (**a**): ESBL, harboring ESBL-encoding genes, (**b**): AmpC, harboring AmpC-encoding genes and (**c**): carbapenemase, harboring carbapenemase-encoding genes either in combination (dashed bars) or as single genes (black bars)

The majority of ESBL-producing isolates (*n* = 26/30, 86.7%) co-harbored more than one ESBL-encoding gene versus only 10% of isolates expressed only a single gene. The highest co-existence patterns were *bla*_TEM_ + *bla*_SHV_ + *bla*_CTX-M-15_ and *bla*_SHV_ + *bla*_CTX-M-15_, represented by 14 and 5 isolates out of 30 ESBL producers, respectively (Fig. 3a).

A correlation matrix between the tested ESBL genes indicated a significant and strongest correlation between *bla*_SHV_ and *bla*_CTX-M-15_ (*rs* = 0.45) (Supplementary Figure S5a).

Among the 20 isolates phenotypically identified as AmpC producers, 19 (95%) were identified as carrying at least one of the detected AmpC genes, in the following order of prevalence: *bla*_AmpC_ (*n* = 16, 80%), *bla*_ACT_ (*n* = 11, 55%), *bla*_ACC_ (*n* = 5, 25%), and *bla*_FOX_ (*n* = 5, 25%). Only one isolate (No. 57) was negative for all tested AmpC genes (Supplementary Table 2).

Molecular evaluation of AmpC-producing isolates indicated that 60% of isolates co-harbored at least two AmpC-encoding genes, showing 4, 3 and 2 gene patterns (5%, 20% and 35%, respectively) (Fig. 3b). A correlation matrix between the AmpC-tested genes don not show any statistically significant correlations between each gene pair (Supplementary Figure S5b)**.**

MHT-positive *P. mirabilis* isolates (*n* = 6) were tested for carbapenemase-encoding genes (*bla*_OXA_, *bla*_KPC_*, bla*_NDM_*, bla*_VIM-1_, and *bla*_VIM-2_). The resistance gene determinants clarified that; 2, 2 and 1 isolates harbored either *bla*_OXA_ or *bla*_VIM-1_ or *bla*_VIM-2_, respectively. While only one isolate (No. 49) co-harbored *bla*_VIM-1_ and *bla*_VIM-2_. On the other hand, *bla*_KPC_ and *bla*_NDM_ were not detected in any of the tested isolates (Supplementary Table 2, Fig. 3c).

### DNA fingerprinting analysis by ERIC-PCR

**Fig. 4:**
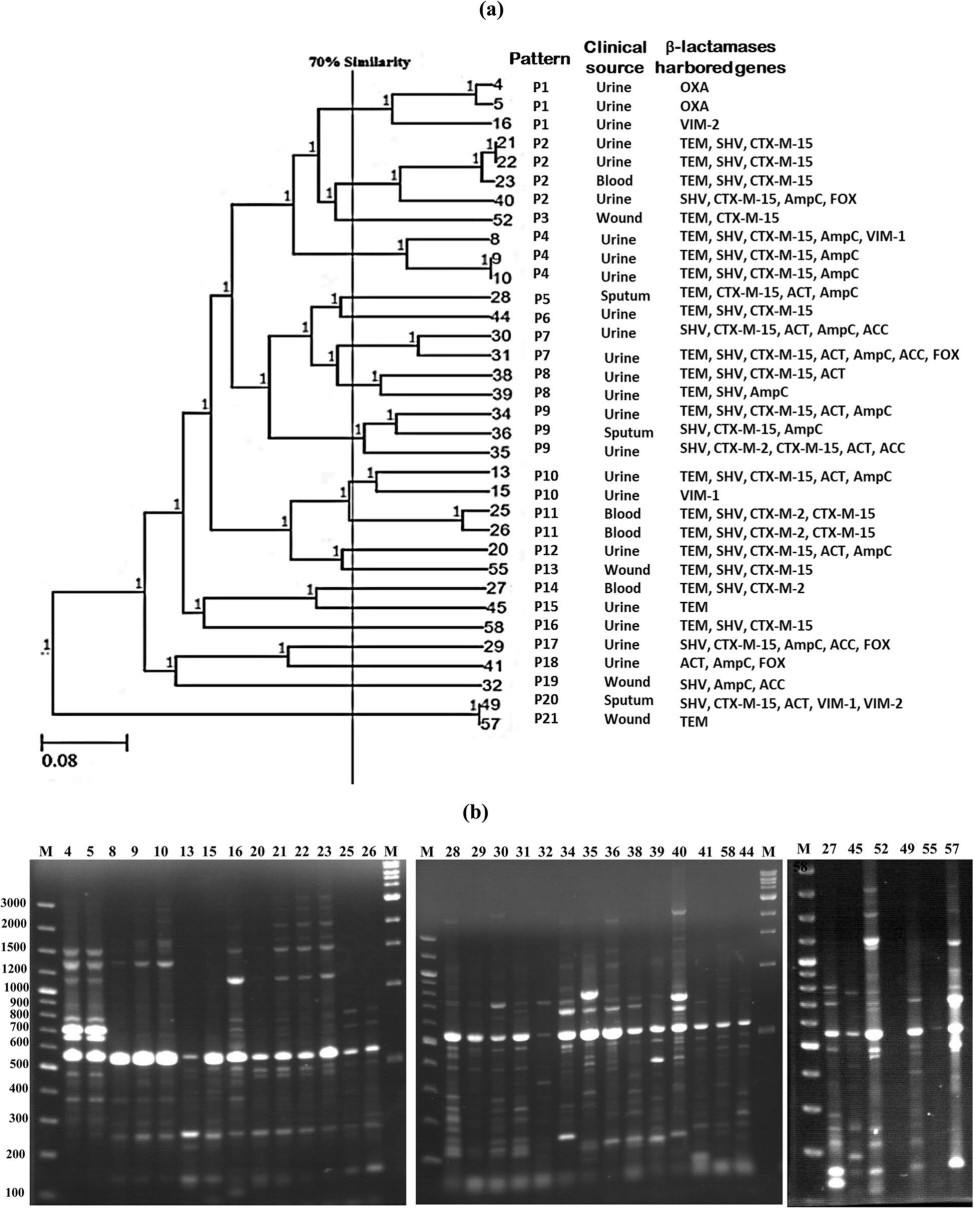
DNA fingerprinting by ERIC-PCR. **a**) Dendrogram analysis of 34 *P. mirabilis* isolates at ≥ 70% similarity using UPGMA based on Dice coefficients method derived from analysis of the ERIC-PCR profiles. Each isolate clinical source and β-lactamase encoding genes pattern are also shown. **b**) Agarose gel electrophoresis of ERIC-PCR for *P. mirabilis* isolates which exhibited Extended Spectrum β-lactamases, AmpC and Carbapenemases using primers ERIC 1 and ERIC 2

Thirty-four (58.6%) *Proteus* isolates were positive for at least one of the tested β-lactamase-encoding genes. The fingerprints obtained from a PCR assay with ERIC primers of these isolates showed a DNA banding profile consisting of amplified bands from 100 to 3000 bp in size (Fig. 4). Based on the binary data of these isolates, a dendrogram (Fig. 4) was constructed. Twenty-one different patterns (P1–P21) were identified among the tested isolates. Isolates were considered to have the same pattern upon a similarity level of ≥ 70%.

All patterns were associated with a low number of isolates, varying from 1 to 4. The predominant pattern was P2 (4 isolates), followed by P1, P4, P9 (3 isolates each), and P7, P8, P10, P11 (2 isolates each). All patterns of isolates were from the same source, with the exceptions of P2 and P9, which were from different sources. Isolates in P1 and P11 were pure carbapenemase and ESBL producers, respectively. Meanwhile, other repetitive patterns (P2, P4, P7, P8, P9, and P10) contained ESBL- and AmpC-producing isolates. Finally, 100% ERIC typing similarities were found between isolates 21 and 22 (in P2), and 9 and 10 (in P4).

## Discussion

The genus *Proteus* is one of the resident microbiota in the human gastrointestinal tract. It is an inherent cause of community-acquired infections and ranks third as a cause of hospital-associated cases [30, 31]. *Proteus mirabilis* is one of the species most commonly isolated from the urine since it is a common causative agent of catheter-associated UTIs [3, 32].

In the present study, 58 *P. mirabilis* isolates were isolated from various clinical samples: urine, wound, blood, sputum, and CSF. The highest percentage of isolates was from urine (69%), followed by wound specimens (13.8%). The ability of *P. mirabilis* to produce urease and accumulate ammonia leads to the creation of an environment where it can survive, colonize the urinary tract, and increase the risk of pyelonephritis and upper UTIs [33].

In the past, the majority of *P. mirabilis* isolates were susceptible to common classes of antibiotics, but recently the emergence of antibiotic resistance has been increasingly reported [34]. In this study, the highest rate of resistance was to cotrimoxazole (**60.3**%), which is similar to the rate in a study conducted in Iran [34, 35] and China [36]. On the other hand, imipenem showed the lowest rate of resistance (**1.7**%) among the tested antibiotics, as also previously detected in India [37]. Moreover, slightly low resistance was detected against aztreonam (**13.8**%) compared with the level previously detected in Egypt [2], suggesting that these antibiotics can be used in the treatment of *P. mirabilis* infections **(**Table 1**)**.

The coexistence of resistance to different antibiotics indicates a simultaneous and continuous transfer of resistance traits among bacterial pathogens and highlights the propensity for MDR to emerge in *P. mirabilis* [4]**.** This study showed a lower MDR level (**29.3**%) than in studies performed in Gondar (87.4%) [38] and Bahir Dar (93.1%) [39] in Ethiopia. However, the magnitude of MDR found here was both very close to Pandey and coauthors [40] and higher than results reported by others [34].

Most of MDR isolates were from urine source (52.9%), indicating that *P. mirabilis* has a higher propensity for colonizing the urinary tract and expressing resistance to different antibiotic classes, especially cephalosporins, fluoroquinolones, and aminoglycosides [41]. The emergence of MDR is a cause of concern that requires the establishment of strong infection control strategies to reduce this burden [42]. The MARI value obtained ranged from 0.27 to 0.82 (Table 2), which significantly exceeds the maximum MARI value calculated at 0.2 [43]. This high MARI values suggest that the tested antibiotics are being used intensively in the area of sample collection and implies an environment with a high risk of antimicrobial resistance proliferation [44].

AmpC β-lactamase-producing isolates are the major cause of nosocomial outbreaks and treatment failure [45]. In this study, 28 isolates were not susceptible to cefoxitin; however, confirmation by a cloxacillin inhibitor-based test showed that 20 (34.5%) isolates were positive for AmpC production (Fig. 1a, Supplementary Figure S2). A study performed in India (37%) [46] showed equivalent levels; however, studies performed in Iran (1.5%) [47] and Spain (14.2%) [48] showed a low incidence of AmpC production. Eight *P. mirabilis* isolates were not susceptible to cefoxitin and did not produce AmpC. This may be attributable to other resistance mechanisms, such as porin channel mutation [49]. On the other hand, some of the AmpC-producing isolates (7 isolates) showed in vitro sensitivity to amoxicillin/clavulanic acid, but the therapeutic use of this antibiotic is doubtful due to AmpC not being inhibited by β-lactamase inhibitors [50].

The current study also demonstrated the coexistence of AmpC with ESBL enzymes since all of the 20 AmpC-producing isolates were also ESBL-positive (Fig. 1b), as reported at low levels in India and Ghana [37, 51, 52]. The effect of ESBLs can be blocked by the overproduction of plasmid-mediated AmpC β-lactamase enzymes [53]. Therefore, simultaneous detection of ESBLs, in the presence of AmpC inhibitor, is important to prevent the possibility of false-negative results.

The overall rate of ESBL-producing *P. mirabilis* in the present study was 51.7% (30 isolates) (Fig. 1a, Supplementary Figure S3), which is in line with other studies [39, 54–56]. In contrast, our prevalence rate was higher than that reported in earlier Egyptian studies, namely, 17% by [57] and 38.8% by [58]. Therefore, an increase in the ESBL production rate has emerged over time in Egypt due to the uncontrolled use of antimicrobials, mostly third-generation cephalosporins, as routine therapy against UTIs [59, 60].

Concerning the relation between in vitro antimicrobial resistance profiles and β-lactamase production (Fig. 2), this study showed that β-lactamase-producing isolates had significantly higher resistance to most of antibiotics tested compared to non-β-lactamase producers. Furthermore, the rate of MDR in β-lactamase producers (88.2%) was significantly higher than that in non-β-lactamase producers (11.8%, *p* < 0.0001). These results are quite concerning that implies the undermining of existing antibiotics’ efficacy and could also hinder the development of new ones [44].

At the genotypic level, out of 30 ESBL-producing isolates, 29 (96.7%) isolates were positive for ESBL genes, with *bla*_SHV_ type being the most predominant (Supplementary Table 1). *Bla*_SHV_- and *bla*_TEM_-positive strains are usually reported as hospital-related pathogens [61]; both genes were abundant among our isolates (*n* = 25, 83.3%; and *n* = 22, 73.3%, respectively). In addition, higher frequencies of both genes in our work and in a recent report from our area may indicate that *bla*_SHV_ and *bla*_TEM_ are endemic in our locality [62]. Co-carriage of multiple ESBL genes was detected in 86.7% of our isolates (Fig. 3a). Similar prevalence rates have also been reported in *E. coli*, *K. pneumonia*, and Enterobacteriaceae [62–65].

The molecular detection of *bla* genes conferring resistance to AmpC β-lactamase revealed that *bla*_AmpC_ was the most amplified gene (80%), however, *bla*_ACC_ and *bla*_FOX_ were the least observed genes among our isolates (each 25%) (Supplementary Table 2). Meanwhile, CIT [66] and DHA [67] and FOX genes [68, 69] were commonly detected in previous studies. Seven isolates had only one type of AmpC β-lactamase and there were 12 isolates with the co-occurrence of more than one AmpC gene, whereas the majority of the shared genotypes occurred uniquely (Fig. 3b).

Seven *P. mirabilis* isolates were not susceptible to imipenem (Table 1). MHT was performed on these isolates to clarify their mechanism of resistance, showing that 85.7% (6/7 isolates) were able to produce carbapenemases. PCR analysis showed that *bla*_OXA,_*bla*_VIM-1_, and *bla*_VIM-2_ were the only positive genes (Supplementary Table 2, Fig. 3c). Co-harboring of carbapenemase genes was seen in only one isolate (No. 49), which is also an isolate coproducing ESBLs and AmpC. Generally, KPC and VIM-1 are the most common carbapenemase-encoding genes in *P. mirabilis* isolates [70]. Few studies have revealed the presence of OXA-23 [71] and NDM [72, 73] in *P. mirabilis* isolates. However, the study by Bontron and colleagues revealed that *bla*_VIM-1_ mediated elevated resistance to carbapenems in *P. mirabilis* [74].

Analysis of the genetic diversity of 34 non-repeated positive β-lactamase-producing *Proteus* isolates was carried out by the ERIC-PCR fingerprint method, although wide ranges of other molecular typing techniques are currently available. ERIC-PCR is a recommended technique because it is fast, cheap, easy to use, and provides acceptable data, but it has low reproducibility [75, 76]. Based on ERIC-PCR typing, a dendrogram was created, showing genotypic heterogeneity among the 34 *P. mirabilis* isolates. Twenty-one patterns were detected, including eight patterns repeated among analogous isolates at a rate of ≥ 70%. On the other hand, isolates number 21 and 22 from P2 and isolates number 9 and 10 from P4 had the same gel banding patterns, giving 100% homogeneity (Fig. 4). Regarding the ERIC profile, this indicated clonal similarities among these isolates. Clonally similar isolates were in a relationship with the pattern of β-lactamase-encoding genes that they exhibited. Accordingly, outbreaks of antibiotic resistance may be due to the spread of different β-lactamase genes [77].

## Conclusion

The data obtained in this study revealed a high prevalence of ESBL and AmpC production among *P. mirabilis* clinical isolates, especially urinary isolates, with the co-occurrence of their respective genes in the same isolates. β-lactamase production is an important cause of multiple and extensive drug resistance. Although, most of isolates were resistance to the tested antimicrobials, imipenem and aztreonam are considered the remaining options for controlling and managing *Proteus* pathogenicity. ERIC-PCR-based genotyping indicated that some isolates had a 100% identical banding profile, which proved the rapid dissemination of β-lactamase-encoding genes. The occurrence of multiple β-lactamases in bacterial isolates reinforces the importance of an antimicrobial resistance surveillance system and an effective antibiotic policy. In addition, the need for sanitary procedures is crucial to prevent the increased rate of antibiotic resistance among the isolates in the future.

## Supplementary Information

**Additional file 1: Supplementary Table 1.** Specific amplification primer sets for *Proteus mirabilis* clinical isolates. **Supplementary Table 2.** Genotypic detection of β-lactamases in 34 *Proteus mirabilis* isolates. **Supplementary Figure 1.** Detection of extended spectrum β-lactamase (ESBLs) by Double disc synergy test (DDST) in *P. mirabilis* tested isolates 10, 13, 20, 21, 22, 23, 25, 26, 27, 28, 31,32, 34, 38, 39, 44, 45, 52, 55, and 58; Positive production of ESBLs enzymes was detected by a clear cut enhancement in the inhibition zones around ceftazidime (30 μg) and cefotaxime (30 μg) disks towards amoxicillin–clavulanic acid (20/10 μg) disc, as ‘keyhole. **Supplementary Figure 2.** Detection of AmpC by Cefoxitin-Cloxacillin double disc synergy test (DDST) among *P. mirabilis* tested isolates 8, 9, 10, 13, 20, 23, 28, 29, 30, 31, 32, 34, 35, 36, 38, 39, 40, 41, 49, and 57. An increase in the size of the inhibition zone by ≥ 4 mm of the cefoxitin/cloxacillin compared to the un-supplemented cefoxitin disc is an indication of AmpC production. **Supplementary Figure 3.** Detection of extended spectrum β-lactamase (ESBLs) in AmpC-positive isolates by adding cloxacillin (200 µg/ml) to the sterilized melted agar medium. *P. mirabilis* isolates 8, 9, 29, 30, 35, 36, 40, 41, 49, and 57) were positive for ESBLs. Positive production of ESBLs enzymes was detected by enhancement in the inhibition zones around ceftazidime (30 μg) and cefotaxime (30 μg) discs towards amoxicillin–clavulanic acid (20/10 μg) disk. **Supplementary Figure 4.** Detection of carbapenemases by Modified Hodge test (MHT) in* P. mirabilis* tested isolates 4, 5, 8, 15, 16, and 49, while isolate 18 was negative. The presence of a distorted inhibition zone (clover-leaf shaped) of *E. coli* ATCC 25922 growth towards the meropenem disc was considered as a positive test. **Supplementary Figure 5.** A Correlogram representing correlation coefficients between each pair of the investigated (a) ESBL-encoding genes and (b) AmpC-encoding genes. The color intensity represents Spearman’s rank correlation coefficient (*rs*) value (blue circles are positive correlations and red circles are negative ones). Non-statistically significant correlations are crossed out and only statistically significant ones (*p*-value ≤ 0.05) were considered.

## Data Availability

Datasets generated and analyzed during this study are included in this published article and its supplementary information files. Also, the datasets used and/or analyzed during the current study are available from the corresponding author on reasonable request.

## Abbreviations

*P**. **mirabilis*: *Proteus mirabilis*
UTIs: Urinary tract infections
MDR: Multi-drug resistant
XDR: Extensively drug-resistant
ESBLs: Extended-Spectrum β-lactamases
CSF: Cerebrospinal fluid
PRL: Piperacillin
AMC: Amoxicillin/clavulanic acid
ATM: Aztreonam
IPM: Imipenem
FOX: Cefoxitin
CAZ: Ceftazidime
CTX: Cefotaxime
CIP: Ciprofloxacin
TS: Cotrimoxazole
GM: Gentamicin
AK: Amikacin
MHA: Muller Hinton Aga
DDST: Double-disk synergy test
CC: Cefoxitin-cloxacillin
MHT: Modified Hodge test
ERIC-PCR: Enterobacterial Repetitive Intergenic Consensus-Polymerase Chain Reaction
UPGMA: Unweighted pair group method with arithmetic mean
