# Supplementary material for: Prevalence of extended-spectrum β-lactamases, AmpC, and carbapenemases in Proteus mirabilis clinical isolates
Source: BMC Microbiol. 2022 Oct 11;22:247. doi: 10.1186/s12866-022-02662-3 (PMC9552493; doi:10.1186/s12866-022-02662-3)
Supplement: Supplementary file 1 — Additional file 1: Supplementary Table 1. Specific amplification primer sets for Proteus mirabilis clinical isolates. Supplementary Table 2. Genotypic detection of β-lactamases in 34 Proteus mirabilis isolates. Supplementary Figure 1. Detection of extended spectrum β-lactamase (ESBLs) by Double disc synergy test (DDST) in P. mirabilis tested isolates 10, 13, 20, 21, 22, 23, 25, 26, 27, 28, 31,32, 34, 38, 39, 44, 45, 52, 55, and 58; Positive production of ESBLs enzymes was detected by a clear cut enhancement in the inhibition zones around ceftazidime (30 μg) and cefotaxime (30 μg) disks towards amoxicillin–clavulanic acid (20/10 μg) disc, as ‘keyhole. Supplementary Figure 2. Detection of AmpC by Cefoxitin-Cloxacillin double disc synergy test (DDST) among P. mirabilis tested isolates 8, 9, 10, 13, 20, 23, 28, 29, 30, 31, 32, 34, 35, 36, 38, 39, 40, 41, 49, and 57. An increase in the size of the inhibition zone by ≥ 4 mm of the cefoxitin/cloxacillin compared to the un-supplemented cefoxitin disc is an indication of AmpC production. Supplementary Figure 3. Detection of extended spectrum β-lactamase (ESBLs) in AmpC-positive isolates by adding cloxacillin (200 µg/ml) to the sterilized melted agar medium. P. mirabilis isolates 8, 9, 29, 30, 35, 36, 40, 41, 49, and 57) were positive for ESBLs. Positive production of ESBLs enzymes was detected by enhancement in the inhibition zones around ceftazidime (30 μg) and cefotaxime (30 μg) discs towards amoxicillin–clavulanic acid (20/10 μg) disk. Supplementary Figure 4. Detection of carbapenemases by Modified Hodge test (MHT) in P. mirabilis tested isolates 4, 5, 8, 15, 16, and 49, while isolate 18 was negative. The presence of a distorted inhibition zone (clover-leaf shaped) of E. coli ATCC 25922 growth towards the meropenem disc was considered as a positive test. Supplementary Figure 5. A Correlogram representing correlation coefficients between each pair of the investigated (a) ESBL-encoding genes and (b) AmpC-encoding genes. The [file 12866_2022_2662_MOESM1_ESM.pdf]

Supplementary Material

Prevalence of Extended Spectrum β-lactamases, AmpC and Carbapenemases in *Proteus mirabilis* Clinical Isolates

Mona Shaaban [*1†], Soha Lotfy Elshaer [1], Ola A Abd El-Rahman [2†]

[1] Department of Microbiology and Immunology, Faculty of Pharmacy, Mansoura University, Mansoura 35516, Egypt

[2] Department of Microbiology and Immunology, Faculty of Pharmacy (Girls), Al-Azhar University, Cairo 11651, Egypt

[*]Corresponding author

Dr. Mona Shaaban

Mona_ibrahem@mans.edu.eg

Dr. Soha Lotfy Elshaer

dr_sohalotfyeldamarawy@mans.edu.eg

[†]These authors contributed equally to this work

Keywords: *Proteus mirabilis*, Extended spectrum β-lactamases, AmpC β-lactamases, Carbapenemases, ERIC-PCR

Supplementary Table 1: Specific amplification primer sets for *Proteus mirabilis* clinical isolates

Gene type	Gene Name	Type of primer	Primer Sequence-3'	MT	Ampli-con size (bp)
ESBLs	*bla*TEM	Fw	5`– GATCTCAACAGCGGTAAG–3`	55°C	786
		Rev	5`– CAGTGAGGCACCTATCTC–3`		
	*bla*SHV	Fw	5`– ACTATCGCCAGCAGGATC–3`	58°C	356
		Rev	5`– ATCGTCCACCATCCACTG–3`		
	*bla*CTX-M-2	Fw	5`– GATGACTCAGAGCATTCG–3`	55°C	739
		Rev	5`– GTTGGTGGTGCCATAATC–3`		
	*bla*CTX-M-15	Fw	5`– GTGATACCACTTCACCTC–3`	54°C	255
		Rev	5`– AGTAAGTGACCAGAATCAG–3`		
AmpC	*bla*ACT-1	Fw	5`– CATGCTGGATCTGGCAACCT–3`	60°C	343
		Rev	5`– CTTCAGCGTCCAGCATTCC–3`		
	*bla*AmpC	Fw	5`– ACACGAGTTTGCATCGCCTG–3`	58°C	254
		Rev	5`– CTGAACTTACCGCTAAACAGTGGA AT–3`		
	*bla*ACC-1	Fw	5`– AGCTGTTATCCGTGATTACCTGTCT–3`	60°C	248
		Rev	5`– AGCGAACCCACTTCAAATAACG –3`		
	*bla*FOX-1	Fw	5`– GCAAACCAGCAATACCATCCA–3`	58°C	642
		Rev	5`– GCTCACCTTGTCATCCAGCTC–3`		
Carbapene-mases	*bla*NDM-1	Fw	5`– ATGCACCCGGTCGCGAAGC –3`	60 ºC	795
		Rev	5`– TCAGCGCAGCTTGTCGGCC –3`		
	*bla*KPC	Fw	5`– ATTCGCTAAACTCGAACAG –3`	50°C	130
		Rev	5`– AAGAAAGCCCTTGAATGAG –3`		
	*bla*VIM-1	Fw	5`– TGTTATGGAGCAGCAACGATG –3`	55°C	920
		Rev	5`– AAAGTCCCGCTCCAACGATT –3`		
	*bla*VIM-2	Fw	5`– GTCTATTTGACCGCGTCTATC–3`	55°C	774
		Rev	5`– CTACTCAACGACTGAGCGAT–3`		
	*bla*OXA	Fw	5`– AAGTGTGCAACGCAAATGGC –3`	55°C	137
		Rev	5`– CTGTTCCAGATCTCCATTCC –3`		

FW: forward Rev: reverse MT: melting temperature bp: base pair

Supplementary Table 2: Genotypic detection of β-lactamases in 34 *Proteus mirabilis* isolates.

Isolate number	Clinical source	*bla*$_{CTX-M-15}$	*bla*$_{CTX-2}$	*bla*$_{SHV}$	*bla*$_{TEM}$	*bla*$_{FOX}$	*bla*$_{ACC}$	*bla*$_{AmpC}$	*bla*$_{ACT}$	*bla*$_{VIM-1}$	*bla*$_{VIM-2}$	*bla*$_{NDM}$	*bla*$_{KPC}$	*bla*$_{OXA}$
4	Urine	-	-	-	-	-	-	-	-	-	-	-	-	+
5	Urine	-	-	-	-	-	-	-	-	-	-	-	-	+
8	Urine	+	-	+	+	-	-	+	-	+	-	-	-	-
9	Urine	+	-	+	+	-	-	+	-	-	-	-	-	-
10	Urine	+	-	+	+	-	-	+	-	-	-	-	-	-
13	Urine	+	-	+	+	-	-	+	+	-	-	-	-	-
15	Urine	-	-	-	-	-	-	-	-	+	-	-	-	-
16	Urine	-	-	-	-	-	-	-	-	-	+	-	-	-
20	Urine	+	-	+	+	-	-	+	+	-	-	-	-	-
21	Urine	+	-	+	+	-	-	-	-	-	-	-	-	-
22	Urine	+	-	+	+	-	-	-	-	-	-	-	-	-
23	Blood	+	-	+	+	+	-	+	+	-	-	-	-	-
25	Blood	+	+	+	+	-	-	-	-	-	-	-	-	-
26	Blood	+	+	+	+	-	-	-	-	-	-	-	-	-
27	Blood	-	+	+	+	-	-	-	-	-	-	-	-	-
28	Sputum	+	-	-	+	-	-	+	+	-	-	-	-	-
29	Urine	+	-	+	-	+	+	+	-	-	-	-	-	-
30	Urine	+	-	+	-	-	+	+	+	-	-	-	-	-
31	Urine	+	-	+	+	+	+	+	+	-	-	-	-	-
32	Wound	-	-	+	-	-	+	+	-	-	-	-	-	-
34	Urine	+	-	+	+	-	-	+	+	-	-	-	-	-
35	Urine	+	+	+	-	-	+	-	+	-	-	-	-	-
36	Sputum	+	-	+	-	-	-	+	-	-	-	-	-	-
38	Urine	+	-	+	+	-	-	-	+	-	-	-	-	-
39	Urine	-	-	+	+	-	-	+	-	-	-	-	-	-
40	Urine	+	-	+	-	+	-	+	-	-	-	-	-	-
41	Urine	-	-	-	-	+	-	+	+	-	-	-	-	-
44	Urine	+	-	+	+	-	-	-	-	-	-	-	-	-
45	Urine	-	-	-	+	-	-	-	-	-	-	-	-	-
49	Sputum	+	-	+	-	-	-	-	+	+	+	-	-	-
52	Wound	+	-	-	+	-	-	-	-	-	-	-	-	-
55	Wound	+	-	+	+	-	-	-	-	-	-	-	-	-
57	Wound	-	-	-	+	-	-	-	-	-	-	-	-	-
58	Urine	+	-	+	+	-	-	-	-	-	-	-	-	-

+: Positive, -: Negative

Supplementary Figures

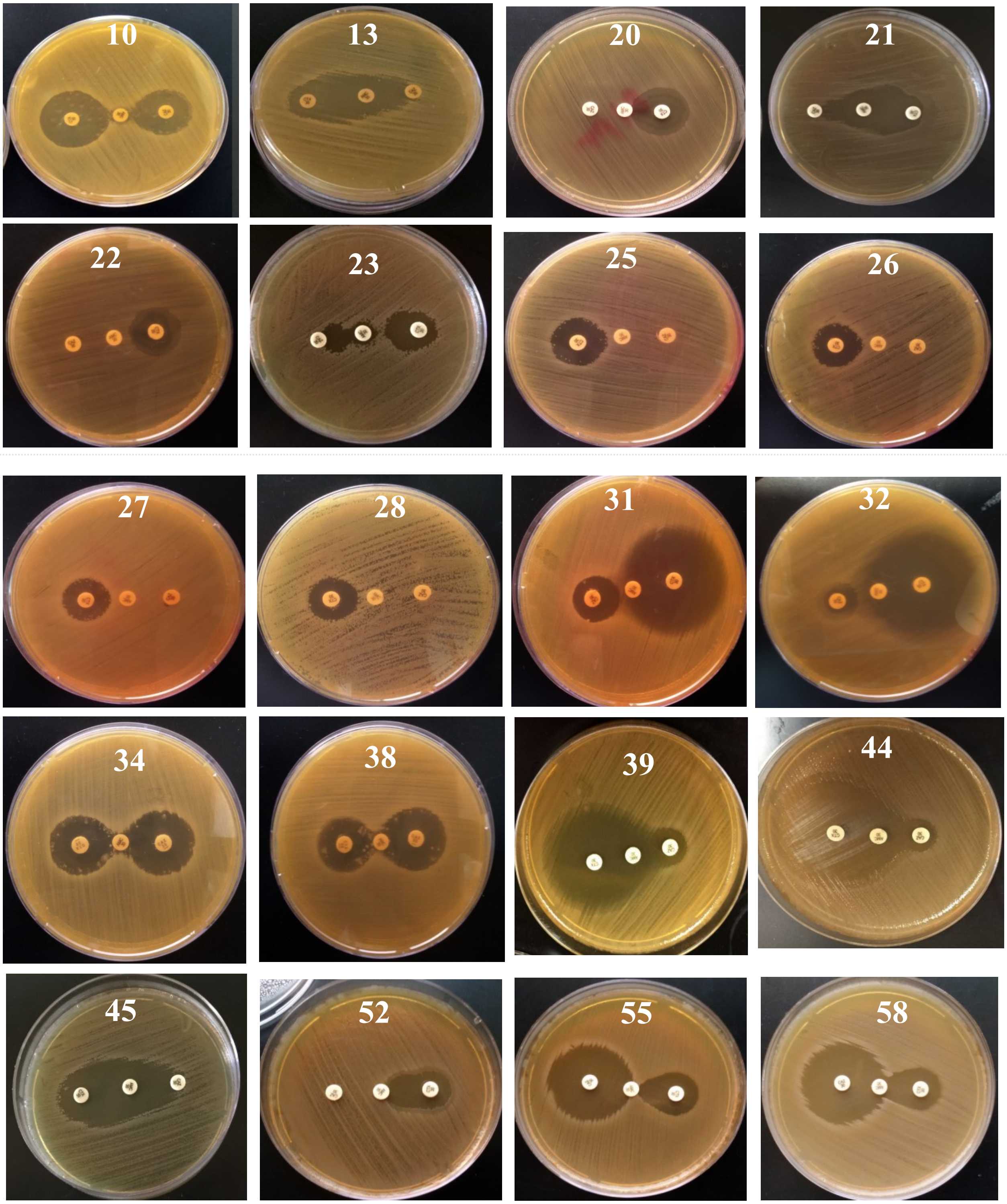

Supplementary Figure 1. Detection of extended spectrum β-lactamase (ESBLs) by Double disc synergy test (DDST) in *P. mirabilis* tested isolates 10, 13, 20, 21, 22, 23, 25, 26, 27, 28, 31,32, 34, 38, 39, 44, 45, 52, 55, and 58; Positive production of ESBLs enzymes was detected by a clear cut-enhancement in the inhibition zones around Ceftazidime (30 µg) and cefotaxime (30 µg) disks towards amoxicillin–clavulanic acid (20/10 µg) disc, as ‘keyhole.

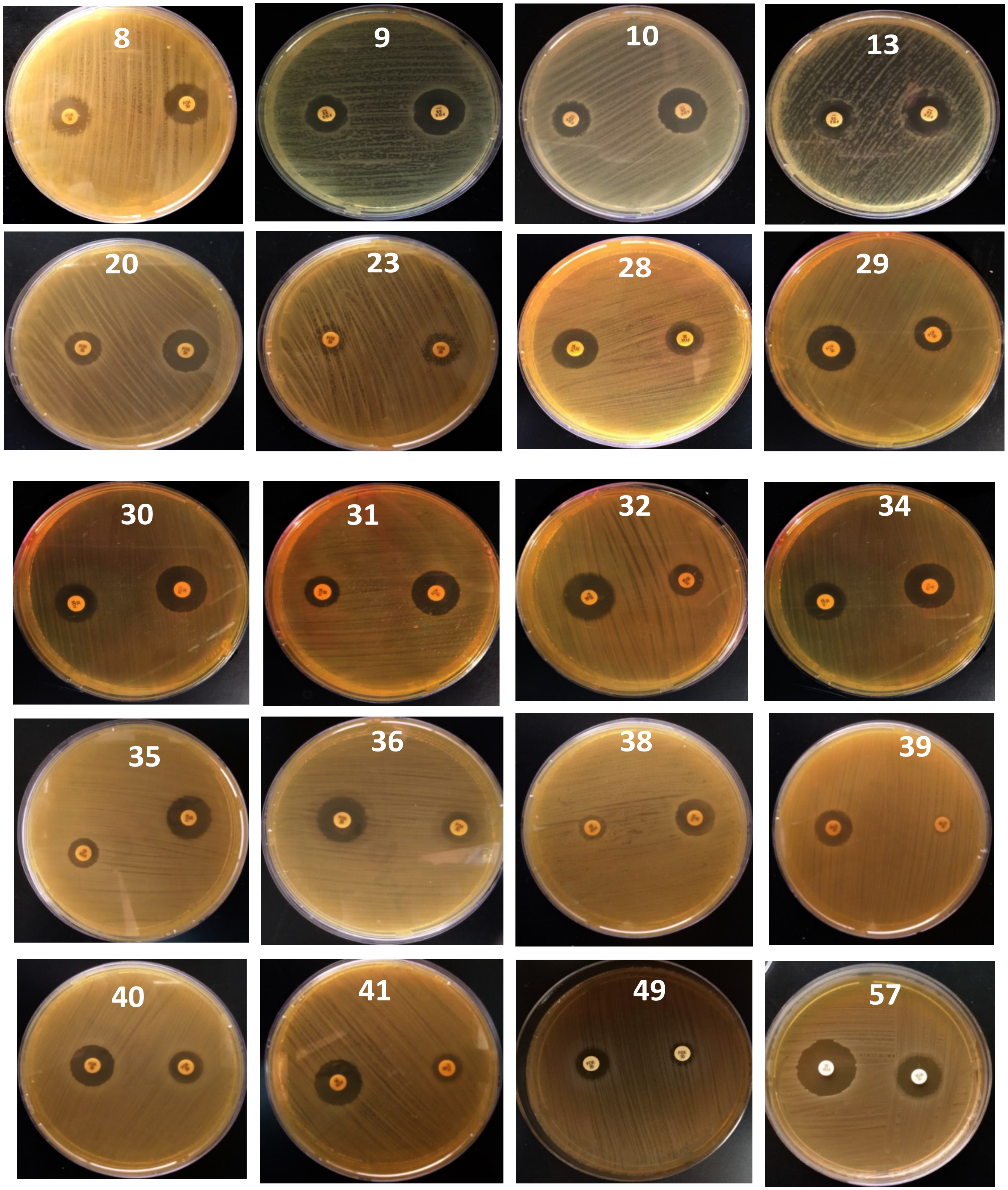

Supplementary Figure 2. Detection of AmpC by Cefoxitin-Cloxacillin double disc synergy test (DDST) among *P. mirabilis* tested isolates 8, 9, 10, 13, 20, 23, 28, 29, 30, 31, 32, 34, 35, 36, 38, 39, 40, 41, 49, and 57. An increase in the size of the inhibition zone by ≥ 4 mm of the cefoxitin/cloxacillin compared to the un-supplemented cefoxitin disc is an indication of AmpC production.

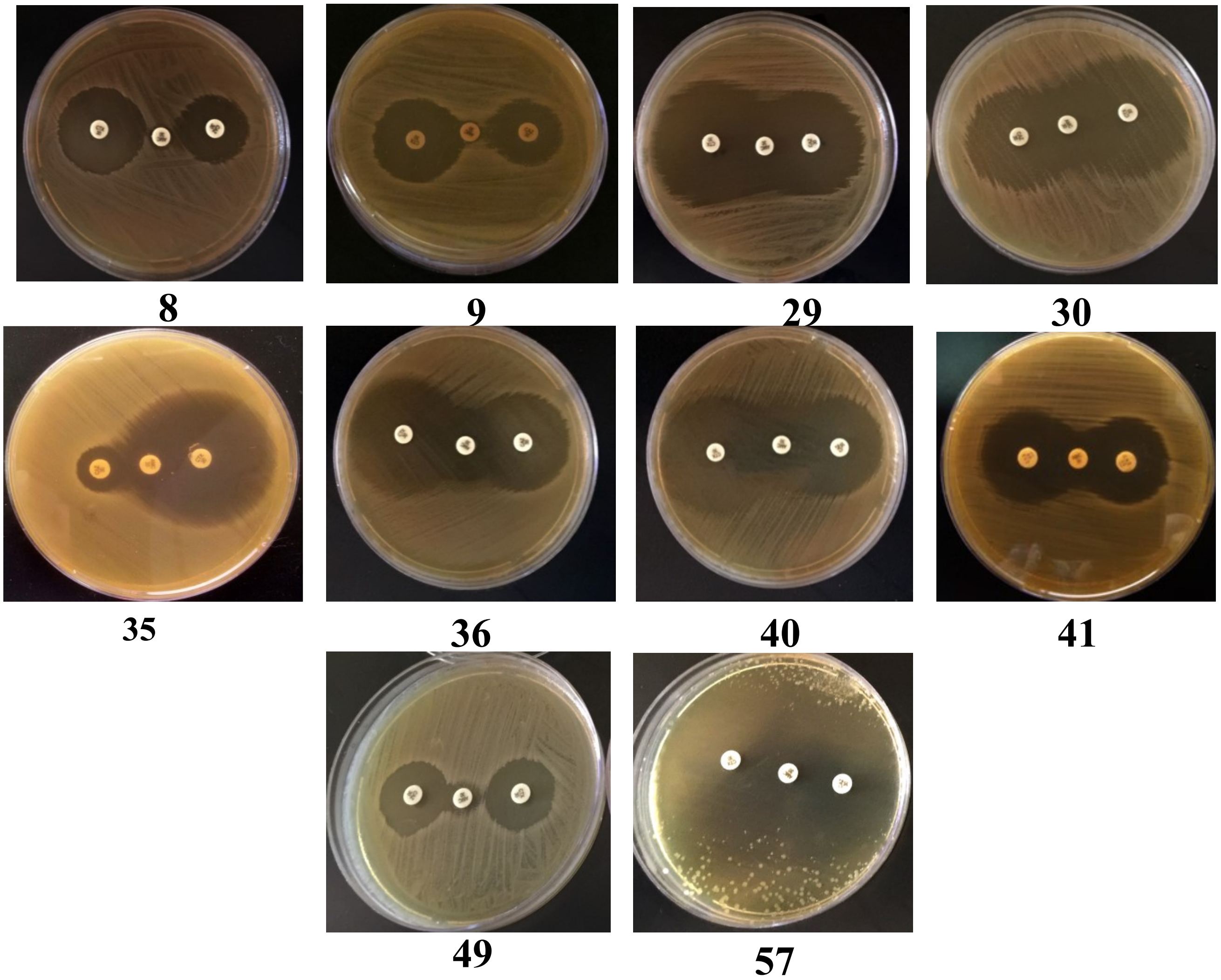

Supplementary Figure 3. Detection of extended spectrum β-lactamase (ESBLs) in AmpC-positive isolates by adding cloxacillin (200 µg/ml) to the sterilized melted agar medium**. *P. mirabilis* isolates** 8, 9, 29, 30, 35, 36, 40, 41, 49, and 57) were positive for ESBLs. Positive production of ESBLs enzymes was detected by enhancement in the inhibition zones around Ceftazidime (30 µg) and cefotaxime (30 µg) discs towards amoxicillin–clavulanic acid (20/10 µg) disk

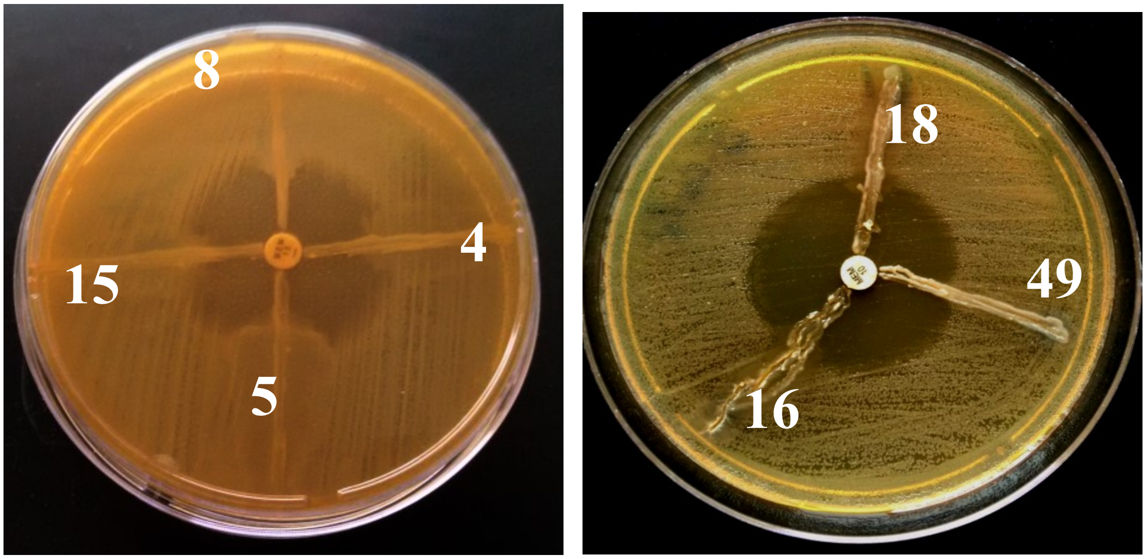

Supplementary Figure 4. Detection of carbapenemases by Modified Hodge test (MHT) in *P. mirabilis* tested isolates 4, 5, 8, 15, 16, and 49, isolate 18 was negative. The presence of a distorted inhibition zone (Clover-leaf shaped) of *E. coli* ATCC 25922 growth towards the meropenem disc was considered as a positive test

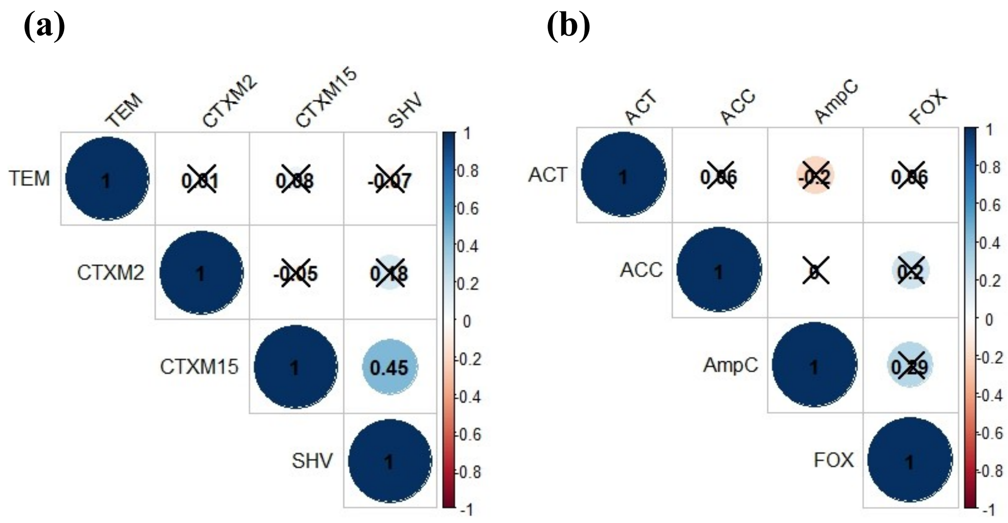

Supplementary Figure 5. A Correlogram representing correlation coefficients between each pair of the investigated (a) ESBL-encoding genes and (b) AmpC-encoding genes. The color intensity represents Spearman's rank correlation coefficient (*rs*) value (blue circles are positive correlations and red circles are negative ones). Non-statistically significant correlations are crossed out and only statistically significant ones ($p\text{-}value \leq 0.05$) were considered.
